# Hydrophilic surface modification of PDMS for droplet microfluidics using a simple, quick, and robust method via PVA deposition

**DOI:** 10.1038/micronano.2016.91

**Published:** 2017-04-24

**Authors:** Tatiana Trantidou, Yuval Elani, Edward Parsons, Oscar Ces

**Affiliations:** 1 Department of Chemistry, Imperial College London, London SW7 2AZ, UK; 2 Institute of Chemical Biology, Imperial College London, London SW7 2AZ, UK; 3 London Centre for Nanotechnology, University College London, London WC1E 6BT, UK

**Keywords:** double emulsions, droplet microfluidics, hydrophobic, hydrophilic, PDMS, surface modification

## Abstract

Polydimethylsiloxane (PDMS) is a dominant material in the fabrication of microfluidic devices to generate water-in-oil droplets, particularly lipid-stabilized droplets, because of its highly hydrophobic nature. However, its key property of hydrophobicity has hindered its use in the microfluidic generation of oil-in-water droplets, which requires channels to have hydrophilic surface properties. In this article, we developed, optimized, and characterized a method to produce PDMS with a hydrophilic surface via the deposition of polyvinyl alcohol following plasma treatment and demonstrated its suitability for droplet generation. The proposed method is simple, quick, effective, and low cost and is versatile with respect to surfactants, with droplets being successfully generated using both anionic surfactants and more biologically relevant phospholipids. This method also allows the device to be selectively patterned with both hydrophilic and hydrophobic regions, leading to the generation of double emulsions and inverted double emulsions.

## Introduction

Polydimethylsiloxane (PDMS) is considered by many to be the material of choice for the fabrication of microfluidic devices^1–3^. Its wide adoption has been responsible in large part for the proliferation of microfluidics in the last two decades. Its attractiveness as a material is due to a wide and varied set of advantages that include low cost, chemical inertness, non-toxicity, and the ability to translate features in the micrometer range^3^. In addition, it is optically transparent and permeable to gases^4^. It is an elastic and physically robust material that is reversibly deformable, and it can be used to make components, including valves and pumps, in microfluidic devices^5^. The fabrication of devices using PDMS soft lithography is simpler, cheaper, and less time consuming than other competing techniques (e.g., silicon and plastic micromachining).

PDMS is a hydrophobic material (water contact angle >100°)^6^, which has consequences for droplet-based microfluidics. To successfully generate droplets, the continuous (i.e., external) phase needs to effectively wet the device walls; therefore, PDMS is ideally suited for the generation of water-in-oil (w/o) droplets^7^. However, the hydrophobicity of PDMS prevents the production of oil-in-water (o/w) droplets with native, untreated PDMS. The generation of o/w droplets is key for various microfluidic applications, including the synthesis of advanced nano- and micro-materials in oil droplet microreactors^8,9^, organic functional group transformations^10,11^, encapsulation of single cells in double emulsions followed by flow cytometric sorting^12^, and the formation of vesicles^13^ and other model membranes^14,15^. The incompatibility of PDMS in this regard has led researchers to use alternative, less desirable materials, such as glass and silicon^16–18^.

In response to this, there have been efforts to modify the PDMS surface to become hydrophilic. These efforts have included chemical vapor deposition of polymer coatings^19^, incorporation of an amphiphilic surfactant in the PDMS bulk^20^, deposition of glass-like layers on the substrate surface (sol-gel coating)^21^, and layer-by-layer (LbL) deposition of charged polyanions and polycations^22^. One well-established approach to produce hydrophilic PDMS surfaces is to oxidize the polymer surface with plasma or ultraviolet (UV) irradiation^23^. However, this effect is transient, and the hydrophobic nature of PDMS returns several minutes after plasma or UV exposure because of the migration of the uncured hydrophobic polymer chains to the surface. Methods to slow down or prevent this recovery from occurring include keeping the surface in water immediately after treatment and removing uncured polymers using solvent extraction^24,25^. However, the utility of these approaches for droplet microfluidics has not been established, and it is likely that exposure of the surface to oil will negate these effects. Another strategy to achieve surface modification is to coat substrates with polyvinyl alcohol (PVA; a hydrophilic polymer). This strategy has primarily been used for non-PDMS surfaces, such as silica capillaries for biopolymer separation and DNA sizing applications^26–28^. PVA can also be irreversibly adsorbed onto hydrophobic polymer films^29^ and gold substrates^30^. Others have demonstrated PVA adsorption onto PDMS via heat immobilization following plasma treatment^27^, via plasma oxidation and covalent attachment of an (3-aminopropyl)triethoxysilane (APTES) linker^31^, and via the synthesis of a PVA/PDMS copolymer microsuspension^32^. These procedures were developed to control the degree of biomolecule interaction with the PDMS substrate and not to modify the hydrophobicity of the surface with regards to droplet microfluidics. A combination of PVA and glycerol coating was immobilized on a plasma-treated PDMS microfluidic chip to encourage poly(L-lactic acid) microsphere generation^33^. However, this study relied on a continual supply of PVA during microsphere generation.

The generation process for lipid-stabilized droplets is the most challenging type of droplet generation because of the very specific favorable wettability between lipids and PDMS. There is one elegant example of using PVA-modified surfaces in droplet microfluidics for the controlled generation of lipid vesicles^34^. Although the aforementioned examples demonstrate the potential of the PVA deposition strategy for chemistry and biology, a systematic characterization and optimization of this technique for droplet microfluidics is lacking, specifically regarding the effects of the various process parameters on the surface properties. This has hindered the wide scale adoption of this versatile technique by the microfluidics community, particularly in the field of bottom-up synthetic biology for artificial cell manufacturing.

In the context of droplet microfluidics, several criteria need to be considered when assessing the suitability of the surface modification technique. The surfaces should be sufficiently hydrophilic to generate o/w droplets. The treatment needs to be long lasting and irreversible, especially when the surface is in contact with both oil and water phases. The treatment should be versatile enough to yield droplets with a range of surfactants, including lipids, which are typically considered less effective for stabilizing droplets. Given the increased use of droplets for the construction of artificial membranes, the development of an adequate treatment is of great importance^13–15^. In addition, the modified channel should be biocompatible, and any deposited material should not interfere with cells and biological components. Furthermore, the technique should allow for modifications of different regions on the same device to create both hydrophobic and hydrophilic areas. This is especially relevant in droplet microfluidics, where the phase that is exposed to the channels (the external phase) is different in different regions of the chip (e.g., in double emulsions). This has been achieved by connecting together several devices with differing surface properties^35,36^ and by selectively exposing regions to plasma using a scanning radial microjet^37^. The latter approach is problematic because it necessitates the use of expensive and specific equipment and suffers from hydrophobic recovery issues. A versatile method for selective modification is to use flow through solutions through defined regions of the device^22,36^ and selectively modify these regions. Most importantly, the technique must be practical; it should be simple, low cost, and not time consuming. Although existing surface modification techniques meet some of the criteria above, there is not a single technique that meets all of them. In this article, we developed and optimized an effective, simple, quick, versatile, and cheap method to make long-lasting, hydrophilic PDMS surfaces for droplet microfluidics. The method was based on coating channels with PVA immediately after plasma treatment. We characterized the effects of the procedure on the contact angle and hydrophilic surface lifetime and showed that the PVA-treated devices can generate droplets with traditional surfactants as well as phospholipids. The procedure can pattern specific regions of the device to exhibit defined wettability characteristics, which is further demonstrated in droplet microfluidics for the generation of emulsions, double emulsions, and inverse double emulsions using a single device design.

## Materials and methods

### Chemicals

Silicon wafers (4 in) were purchased from IBD Technologies Ltd. (Wiltshire, UK). Standard microscope glass slides (75×25×1.5 mm) were obtained from VWR (Leicestershire, UK). Acetone and isopropyl alcohol (IPA) were both obtained from Sigma-Aldrich (Dorset, UK). Negative photoresist SU-8 2050 and EC solvent developer solution were obtained from Chestech Ltd. (Rugby, UK). PDMS prepolymer and the curing agent kit (Sylgard 184) were obtained from Dow Corning (Midland, MI, USA). PVA (87–90% and 99+% hydrolysis degree) was purchased from Sigma-Aldrich. The phospholipids, 1-palmitoyl-2-oleoyl-*sn*-glycero-3-phosphocholine (POPC) and 1,2-dioleoyl-*sn*-glycero-3-phosphocholine (DOPC), were obtained from Avanti Polar Lipids (Alabaster, AL, USA). Oleic acid was obtained from Sigma-Aldrich. Span-80 and sodium dodecyl sulfate (SDS) surfactants were purchased from Sigma-Aldrich. Ethanol and glycerol were obtained from Sigma-Aldrich. Milli-Q (Millipore, Billerica, MA, USA) water was used to prepare aqueous solutions. Sudan Red 7B dye was purchased from Sigma-Aldrich.

### PVA surface modification

To dissolve PVA in water, PVA was added to Milli-Q water (1 wt%) and stirred at room temperature for 40 min. The temperature was then gradually increased to 100 °C, and the solution was stirred for another 40 min. Finally, the temperature was reduced to 65 °C, and the solution was left to stir overnight. The container with the solution was weighed, and water was added to compensate for any losses due to water evaporation.

To treat PDMS for the contact angle measurements, the PDMS pieces (15×15×2 mm) were thoroughly degreased in IPA, blown dry with nitrogen, and dehydrated in an oven at 110 °C for at least 40 min. They were then placed inside a Femto plasma cleaner (Diener Electronic, Ebhausen, Germany) and plasma oxidized under various conditions (power intensity and exposure time) at an oxygen flow of 20 sccm and pressure of 0.67 mbar. The PVA solution was immediately poured onto the plasma-treated surface for 10 min at room temperature, and the pieces were blown dry with nitrogen and heated on a hotplate at 110 °C for 15 min.

The hydrophilic treatment of PDMS for the droplet generation microfluidic chips was performed immediately after plasma bonding of the devices (at 100 W for 1 min, 20 sccm O_2_ flow and 0.65 mbar pressure) by passing the PVA solution into the microchannels using a plastic syringe. The PVA solution was left inside the channels for 10 min at room temperature and was thoroughly removed by blowing pressurized nitrogen through the channels and heating at 110 °C for 15 min to remove any residual moisture. When the lipid was used to stabilize the droplets, this process was repeated three times to achieve a sufficient level of surface hydrophilicity and remove all traces of wetting. Similar to previous findings^27^, we found that this process significantly increased the adsorption of PVA onto the PDMS surfaces. When a surfactant was used, a single layer of PVA was sufficient to produce stable droplets.

To selectively modify the hydrophobicity of the channels of a chip, a plastic syringe and tubing were used to pass air through the hydrophobic channels at 200 μL min^−1^ via a syringe pump. The PVA solution was manually passed through the channels that should be turned hydrophilic using a plastic syringe and tubing. A detailed schematic showing the process is shown in Supplementary Figure S1. In o/w/o devices, the air syringe was placed in the outlet, and the PVA solution was manually passed through inlet #1 via a plastic syringe. In w/o/w devices, the air syringe was placed in inlet #1, and inlet #2 was blocked using a plug. The PVA solution was manually passed through the outlet. In both cases, the PVA solution was only passed through the desired path to ensure selective surface modification. Any PVA residuals were then removed by blowing pressurized nitrogen through the channels. The devices were then heated on a hotplate at 110 °C for 15 min. This process was repeated three times when the device was used for lipid-stabilized double emulsions.

To identify any variations in the size and uniformity of the PDMS microchannels before and after the PVA treatment, a Dektak Stylus profiler (Veeco Instruments Inc, St Ives, UK) (5 μm radius) was used directly on the non-bonded PDMS microfluidic chips. For these experiments, the microchannels of the PDMS microfluidic device were first measured with the profilometer. The device was subsequently plasma oxidized (100 W for 1 min) and pressed continuously against a flat, untreated PDMS surface to temporarily seal the microchannels while flushing the PVA solution. The treatment process was the one described above and was repeated three times. After treatment, the device was separated from the flat PDMS substrate and measured with the profilometer again.

### Contact angle measurements

The evaluation of the surface hydrophilicity of the PVA-treated PDMS was performed via static contact angle measurements using a Drop Shape Analyzer DSA100 (Krüss GmbH, Hamburg, Germany). Three different samples were used for each condition. Deionized water (10 μL) was always dropped at the center of the PDMS piece, and the affinity of the drop for the surface was measured using the circle fitting method. The mean contact angle was extrapolated based on the three distinct samples. Measurements were performed for each sample before treatment, immediately after treatment and up to the 30th day after treatment. All samples were maintained in a standard room environment (20 °C and 30–35% humidity).

### Atomic force microscopy (AFM) measurements and analysis

AFM measurements were conducted in contact mode on a Bruker Multimode 8 equipped with an ‘E’ scanner. MSNL silicon nitride cantilevers were used with a spring constant of 0.1 N m^–1^. Si tips were used (MSNL-10, Bruker, Coventry, UK) with a nominal radius of 2 nm. Measurements were performed on untreated and PVA-coated PDMS surfaces in 50 μL aqueous solutions (consisting of 72% v/v Milli-Q water, 14% v/v ethanol, and 14% v/v glycerol) to replicate the experimental conditions during microfluidic droplet generation. As a roughness metric, we used the root mean squared average of the height deviations taken from the mean image data plane (Equation (1)):

(1)Rq=sumzi2N

### Preparation of solutions for droplet and double emulsion generation

In all droplet generation experiments, the aqueous phase was composed of Milli-Q water (72% v/v), ethanol (14% v/v), and glycerol (14% v/v), and all the components were vortexed to give a turbid mixture. Glycerol was added to the aqueous solution to increase the viscosity of the outer phase to aid droplet breakup.

In o/w droplet generation experiments, the oil phase was composed of 1 mg mL^−1^ DOPC lipid in oleic acid. In the double emulsion experiments, 10 mg mL^−1^ POPC in oleic acid was used. To dissolve the lipids in oil, they were first pre-dissolved in chloroform, which was removed under a stream of nitrogen to give a lipid film. Oleic acid was then added, and the mixtures were sonicated for 60 min to fully dissolve the lipid. In experiments with surfactants, the surfactant was 0.1 wt% Span-80 in oil and 0.5 wt% SDS in water.

### Microfluidic device fabrication

The device designs were patterned on a silicon wafer using standard soft lithography techniques to produce a mold. The PDMS prepolymer and curing agent were then thoroughly mixed in a 10:1 ratio. The mixture was cast onto the silicon mold master, thoroughly degassed in a vacuum and cured overnight in the oven at 65 °C. Thin PDMS slabs (~1 mm) were similarly produced inside flat containers. Glass microscope slides were degreased in acetone inside an ultrasonic bath for 5 min, rinsed with IPA, and blown dry with nitrogen. PDMS slabs were bonded with the microscope slides after oxygen plasma treatment (100 W for 1 min, 20 sccm O_2_ flow, and 0.67 mbar pressure) by manually pressing the two parts together. The microfluidic chips were bonded to the PDMS slabs in the same way.

### Microfluidic generation of droplets and double emulsions

The inlet phases of the microfluidic chips were all injected using 1 mL plastic syringes linked to 1.09 PTFE tubing (Adtech Polymer Engineering Ltd., Stroud, UK). Syringe pumps (Chemyx Inc., Stafford, TX, USA) were used to pump the reagents into the microfluidic chips at controlled flow rates. When surfactants were used, the o/w flow rates were 1 and 5 μL min^−1^, respectively. When lipids were used, the o/w flow rates were 5 and 15 μL min^−1^, respectively.

In the o/w/o devices, the solutions were driven into the device at 1, 8, and 20 μL min^−1^, respectively. For visualization purposes, Sudan Red 7B dye (5 mg mL^−1^) was mixed with the internal oil phase.

In the w/o/w devices, the aqueous phases were inserted into the device via inlets at 1, 5, and 12.5 μL min^−1^, respectively, with Sudan Red 7B dye (0.5 mg mL^−1^) added to the oil phase to allow visualization.

### Data acquisition and analysis

All microfluidic experiments were imaged using an inverted Leica DM IRB microscope (Leica Microsystems Ltd., Milton Keynes, UK). For droplet size measurements, an Olympus IX81 microscope with a Phantom high-speed camera (Vision Research Ltd., Bedford, UK) was used, and the size was calculated using an automated process developed in ImageJ (NIH, Baltimore, MD, USA) and Python (Python Software Foundation, Wilmington, DE, USA). The monodispersity of the droplets was extrapolated from the Gaussian fitting of the histogram as the standard deviation over the average value (coefficient of variation). When this number was smaller than 5%, the droplet generation was assumed to be monodispersed.

## Results

### Surface wettability study of PVA-treated PDMS

**Figure 1:**
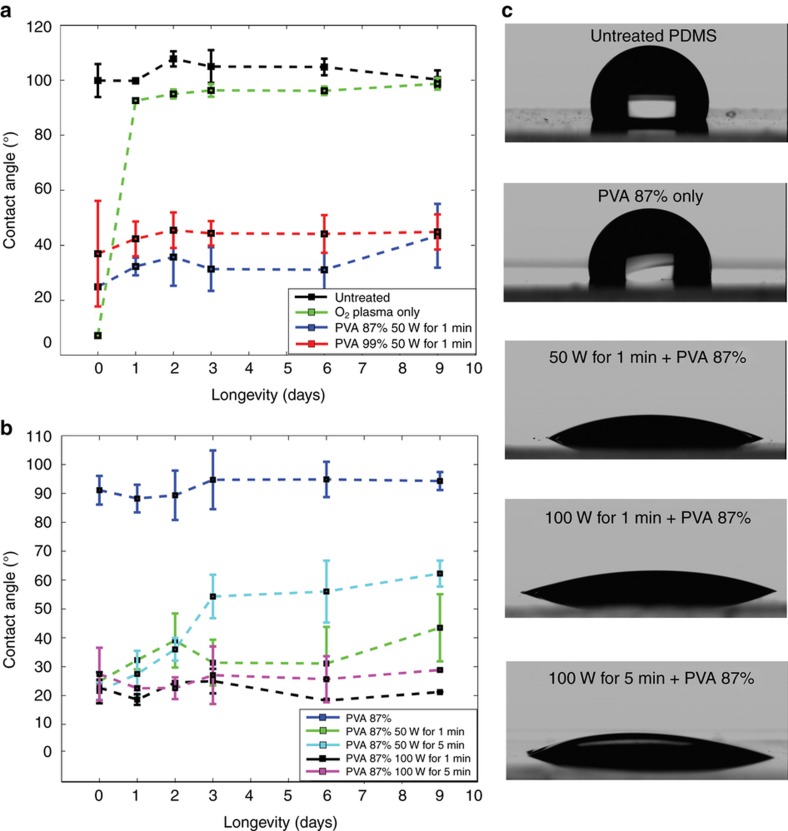
Contact angle measurements of PDMS surfaces as a function of time. (**a**) Untreated and PVA-treated (87–90% and 99+% hydrolysis) PDMS (*N*=3 samples). (**b**) Various oxygen plasma treatment conditions for PVA-treated (87–90%) PDMS (*N*=3 samples). Error bars indicate the standard deviation of the means. (**c**) Affinity of a 10 μL water-in-air droplet for a PDMS surface under various treatment regimes. PDMS, polydimethylsiloxane; PVA, polyvinyl alcohol.

The first step in our treatment was exposing the PDMS surface to oxygen plasma. This treatment generates radical species of surface silanol groups (Si–OH), alcoholic hydroxyls (C–OH), and carboxylic acids (COOH)^27,38,39^ on the PDMS surface and these species can form covalent bonds with the PVA molecules. A series of contact angle measurements were taken to study the effect of PVA deposition on PDMS surface properties (Figure 1). We found that the PDMS treated with plasma and PVA showed significantly lower water–air contact angles than native PDMS (Figure 1a). Moreover, the combined plasma oxidation and PVA treatment resulted in the long-term stable and sustained hydrophilicity of the PDMS surfaces in contrast to the plasma oxidized PDMS, which tended to regain its surface hydrophobicity 1 day after treatment (average contact angle was 7.2±0.5° immediately after plasma oxidation and increased to 92.6±0.3° 1 day later). The complete study on the contact angles of PVA-treated PDMS over a 30-day period is presented in the Supplementary Figure S2.

We evaluated the level of the surface hydrophilicity induced by two commercially available PVA solutions commonly used in PDMS surface modification^27–29,32–34^. We applied PVA solutions with 87–90% and 99+% hydrolysis degrees on PDMS surfaces that were plasma oxidized under the same conditions (50 W for 1 min) and concluded that the former had a more profound effect on the PDMS surface chemistry both in the short and the long term (Figure 1a). The average contact angles for the plasma oxidized PDMS were 24.9±0.4° for PVA 87–90% and 37.0±19.2° for PVA 99+% immediately after treatment, and this difference was retained for 9 days. PVA 87–90% was used for all subsequent experiments. A more detailed study on the effect of PVA 99+% can be found in Supplementary Figure S3.

The oxygen plasma recipe had an important role in the hydrophilic surface modification of PDMS with PVA; plasma treatments at a high power combined with the PVA treatment delivered a more hydrophilic PDMS surface (Figures 1b and c). The highest surface hydrophilicity was achieved at a plasma power value of 100 W (the average contact angles were 22.7±5.4° and 27.5±9.1° for 1- and 5-min treatment durations, respectively), and this power also resulted in a longer-lasting surface hydrophilicity (Figures 1b, c and Supplementary Figure S2). For example, the average contact angle for plasma oxidized PDMS surfaces at 100 W for 1 min was 21.0±3.2° 30 days after treatment.

We found that the surface roughness of the plasma oxidized PDMS surfaces (100 W for 1 min) with a PVA coating was significantly higher than that of the untreated PDMS surfaces (Supplementary Figure S4). The average surface roughness (*R*_*q*_) value for the untreated PDMS was 4.21±0.87 nm (*N*=3 areas) in contrast to the PVA-coated PDMS, which had a value of 14.70±1.73 nm (*N*=3 areas).

### Generation of o/w microdroplets

**Figure 2:**
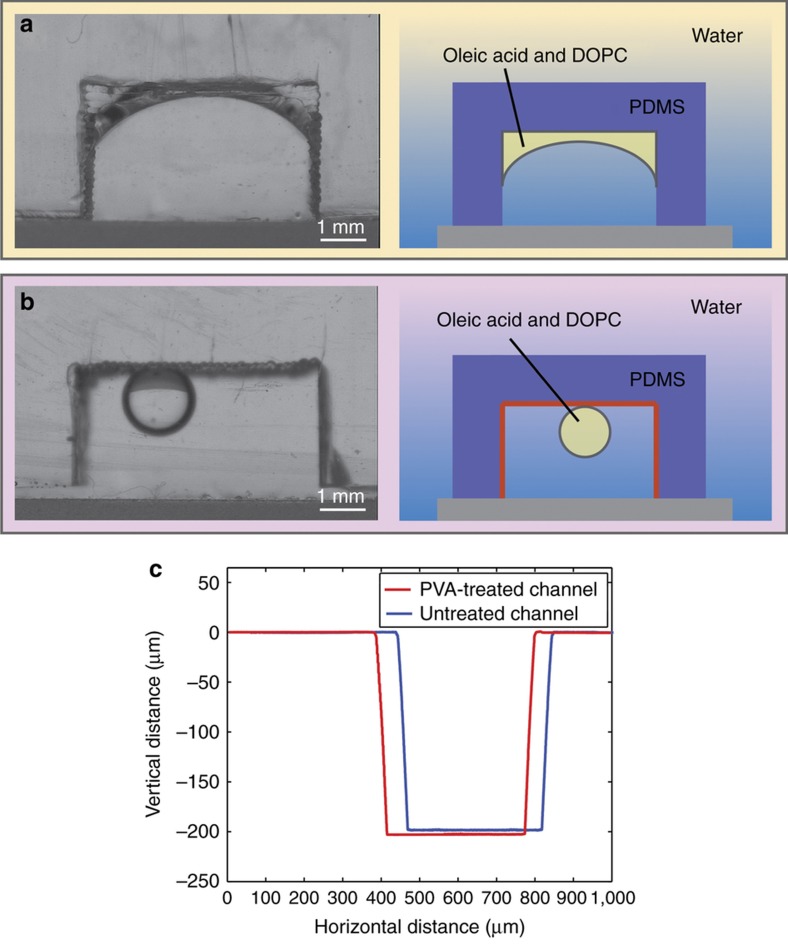
O/w droplet wetting in a PDMS channel. (**a**) Untreated PDMS. Note the severe wetting of the oil phase on the channel walls. (**b**) Plasma oxidized (100 W for 1 min) PDMS treated with PVA (87–90% hydrolysis). Note the lack of wetting by the oil droplet on the hydrophilic channel surface. (**c**) Profilometer scan profiles of a PDMS microchannel (400 μm wide, 200 μm deep): (blue) untreated PDMS device and (red) PVA-treated (100 W for 1 min) PDMS device. PDMS, polydimethylsiloxane; PVA, polyvinyl alcohol.

First, we demonstrated that o/w droplets do not wet the channel surface. For this purpose, we used a larger microfluidic channel (4 mm width×3 mm depth) (Figure 2). Using a syringe, we injected c. 10 μL of oil (oleic acid) containing 1 mg mL^−1^ of DOPC into a PDMS channel containing an aqueous solution. In an untreated PDMS channel, the oil droplet wetted the walls and spread inside the channel (Figure 2a). However, when the PDMS channel was plasma oxidized and PVA coated, the oil droplet remained intact inside the channel (Figure 2b).

The size and uniformity of a PVA-treated channel (400 μm wide and 200 μm deep) was evaluated using a stylus profiler before and after surface modification. The profilometer scan profiles acquired from two distinct channels are presented in Figure 2c. The average values and standard deviations for the vertical distances of the untreated and treated channels were 206.9±3.4 μm (*N*=3 measurements) and 206.9±3.6 μm (*N*=4 measurements). These values correspond to a depth variation in the range of 3.7% from the initial channel depth, which is not significant enough to affect most applications, including droplet and double emulsion generation. We did not observe any variations in the channel’s width based on the horizontal distance data.

**Figure 3:**
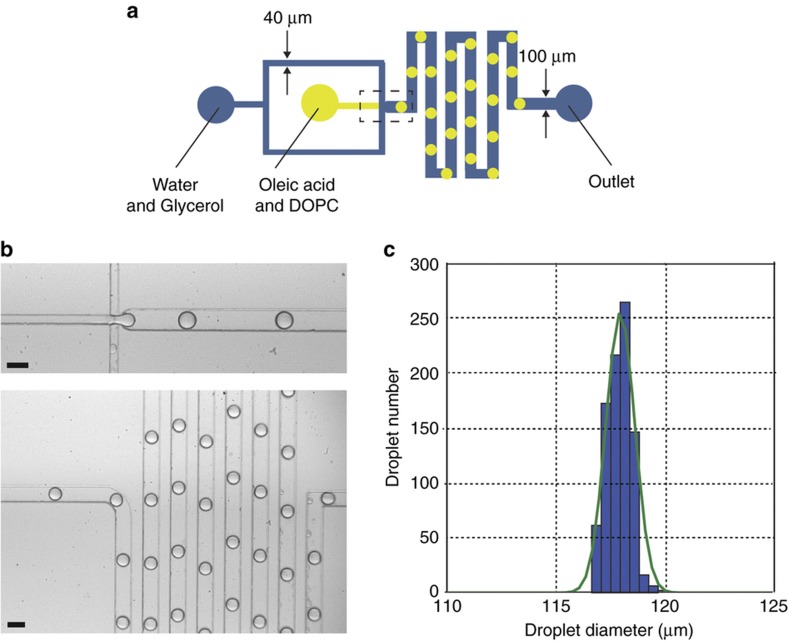
Production of lipid-stabilized o/w droplets using a hydrophilic surface-modified PDMS device that was plasma oxidized (100 W for 1 min) and PVA-coated (87–90% hydrolysis). (**a**) Microfluidic device design. Channel depth is 80 μm. (**b**) Micrographs of the stable formation of o/w droplets with the flow rates of 5 and 15 μL min^−1^, for oil and water respectively. Scale bars=100 μm. (**c**) Diameter distribution of the microfluidic droplets assembled on the chip. The average diameter for this data set (*N*=883 droplets) was 117.9 μm with a coefficient of variation of 0.5%. PDMS, polydimethylsiloxane; PVA, polyvinyl alcohol.

Next, we constructed a PVA-treated microfluidic device based on a flow-focusing geometry for the generation of o/w droplets, and the oil phase contained the DOPC lipid as the surfactant (Figure 3a). The two phases were inserted through two inlets using syringe pumps at flow rates of 15 and 5 μL min^−1^, respectively. The microfluidic device consistently generated smooth droplets at a rate of 570 droplets min^−1^ (Figure 3b and Supplementary Video S1). The oil droplet size distribution was measured (Figure 3c), and the droplets had a high level of droplet monodispersity with a coefficient of variation of 0.5% and an average droplet diameter of 117.9 μm (*N*=883 droplets).

Similarly, the hydrophilic coating was successfully used for oil droplet generation with traditional surfactants instead of lipids. In this case, the two phases were inserted into the chip at flow rates of 1 and 5 μL min^−1^, respectively (Supplementary Video S2). This demonstrated that the proposed modification procedure is equally efficient for surfactant and lipid-oil droplet generation.

Our microfluidic chips were reusable after simply flowing nitrogen through the channels to clear them of the aqueous phases. For the removal of more difficult residues in the channels, such as oils and organic dyes, ethanol was flushed through the channels and followed by a nitrogen flow. This was sufficient to clean the device, which minimizes the effort, time and cost of producing new devices. This cleaning procedure can be repeated several times without affecting the PDMS surface hydrophilicity, and this was supported by the experimental data presented in the Supplementary Figure S5.

To test the long-term efficiency of the proposed treatment, we plasma oxidized and PVA treated a microfluidic device, which was then stored under standard room conditions. The microfluidic chip was tested 30 days after treatment and could still reliably generate lipid/oil droplets (Supplementary Video S3). This showed that the proposed method can reliably deliver off-the-shelf products that can be stored for at least a month without compromising the production of stable and monodisperse emulsions.

### Generation of o/w/o and w/o/w double emulsions

A versatile surface treatment must be capable of being used to construct devices with both hydrophobic and hydrophilic regions via the selective treatment of various areas. In a series of experiments, we used our modification method for the selective modification of defined areas, and we constructed devices capable of generating w/o/w and o/w/o double emulsions. Double emulsions have lately attracted interest for their potential in many applications, such as the encapsulation of small molecules, pesticides, and drugs^40,41^, and they have the potential to serve as small bioreactors for *in situ* production and delivery of pharmacological compounds^42^. Microfluidic production of double emulsions is preferable to bulk preparation methods^43,44^ because it produces monodisperse emulsion droplets and is a very well-controlled process allowing the number of encapsulated droplets to be precisely defined^45^. The production of double emulsions in microfluidic devices is a challenging task as it requires synchronization of the droplet formation frequencies and very specific channel wettability; oil droplet formation can only be realized at a hydrophilic flow-focusing junction, whereas the aqueous droplets can only form at a hydrophobic junction.

**Figure 4:**
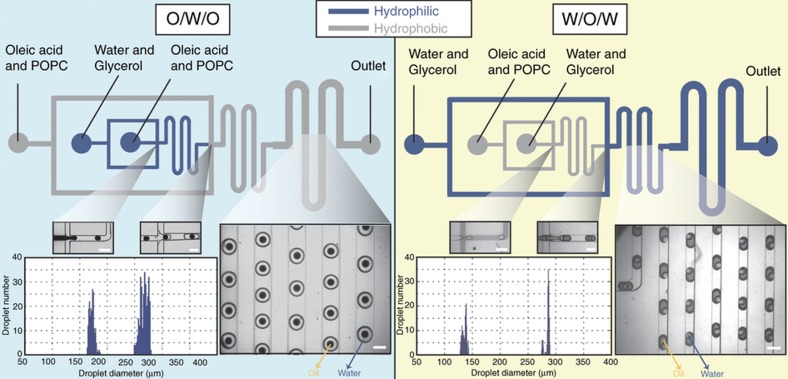
Double emulsion generation on a selectively treated microfluidic device. (Left) O/w/o double emulsions were produced at flow rates of 1, 8, and 20 μL min^−1^, respectively. (Right) W/o/w double emulsions were produced at flow rates of 1, 5, and 12.5 μL min^−1^, respectively. The depths of the channels were 100 and 200 μm at the first and second flow-focusing junctions, respectively. Scale bars=300 μm. In both cases, the diameter distribution shows a high level of droplet monodispersity for the inner and outer droplets.

Here, we demonstrated the versatility of our modification method by precisely patterning the surface chemistry within a network of microchannels. We leveraged the microfluidic device shown in Figure 4 to produce two types of double emulsions: (i) o/w/o double emulsions and (ii) w/o/w double emulsions. These were generated using a microfluidic device consisting of two sequential flow-focusing modules for droplet generation; first to generate the droplets and then to encapsulate the initial droplets in larger droplets. Depending on which channels were modified to be hydrophilic, we were able to produce oil and water droplets at different parts of the microfluidic chip.

To generate o/w/o emulsions, an oil solution was used as both the inner and outer solution, and the middle solution consisted of a water/glycerol mixture (Figure 4, left). Oil droplets were generated at the first junction and were encapsulated by bigger water droplets at the second junction (Supplementary Video S4). One thousand four hundred thirty double emulsions were generated per minute. To increase the visibility, the internal oil phase was dyed with 5 mg mL^−1^ Sudan Red dye. The droplet diameter distribution of both the oil and water droplets was narrow for both the inner oil and the outer water droplets; the average diameter for the oil droplets was 172.3 μm with 2.9% monodispersity (*N*=206 droplets), and the average diameter for the water droplets was 267.2 μm with 2.7% monodispersity (*N*=308 droplets).

For w/o/w generation (Figure 4 right), the water droplets were produced at the first junction and were then encapsulated inside larger oil droplets at the second junction (Supplementary Video S5). To enhance the visibility of the emulsions, Sudan Red dye was added at a lower concentration (0.5 mg mL^−1^) to the middle (oil) phase. The droplet diameter distribution of both the water and oil droplets was narrow for both the inner water and the outer oil droplets; the average diameter for the water droplets was 136.1 μm with 2.9% monodispersity (*N*=168 droplets), and the average diameter for the oil droplets was 285 μm with 1.4% (*N*=152 droplets).

## Discussion

PDMS has dominated the field of microfluidics because of its attractive physical and chemical properties, such as optical transparency, chemical inertness, biocompatibility, simple device fabrication via soft lithography, and easy interfacing of devices with the user. However, its hydrophobicity hinders its use for o/w droplet generation, which is used for many applications in high-end material synthesis in oil droplet microreactors^8,9^ and in more complex multi-phase systems that can serve as advanced microreactors for *in situ* drug synthesis and delivery^40,41^.

Many surface modification techniques have been developed to alter PDMS hydrophobic properties^19–23,37,38^. However, modifying the surface hydrophobicity of PDMS specifically for o/w formation is a challenging process, and despite a contact angle reduction, some surface modification techniques cannot be used for droplet generation^20,23^. This is thought to be because: (i) the channels are not sufficiently hydrophilic, and the wetting of the oil droplets on the device compromises the device operation; (ii) the very process of introducing oil to the channel surface leads to a reversal of the surface treatment. Some treatments, such as plasma oxidation, have only temporary results and the PDMS surfaces restore their natural hydrophobicity a few minutes after the plasma treatment^46^, which was confirmed by our experimental data. Other modification methods, such as LbL deposition, graft photopolymerization, and sol-gel coating, are labor intensive and time consuming, as they require manual injection and removal of solutions, and each step in the method requires several minutes.

In this article, we demonstrated a simple, one-step, robust surface modification method to create hydrophilic microchannels in PDMS-based microfluidic devices, which surpasses several existing techniques. We showed that the PVA surface modification does not suffer from the aforementioned problems and can be used for the generation of o/w droplets and more complex multi-phase systems. The proposed surface modification was achieved by exposing plasma oxidized PDMS surfaces to 1 wt% PVA in water for 10 min. O_2_ plasma creates alcoholic hydroxyls (C–OH), silanols (Si–OH), and carboxylic acids (COOH), which allow hydrogen bonding between the PVA molecules and the activated PDMS surfaces^27^, which leads to permanently hydrophilized surfaces. In addition, a multilayer PVA assembly is facilitated through hydrophobic interactions between the main chain groups (–CH_2_–CH–) and the intermolecular hydrogen bonding of the γ–OH hydroxyl groups between the absorbed PVA after drying out the PDMS surface and the PVA chain in aqueous solution^30^. Our experimental data show that the PVA-treated PDMS surfaces retain their hydrophilicity in the long term. We leveraged PVA solutions with two distinct hydrolysis degrees (87–90% and 99+%) because these are widely used for surface modification in PDMS-based microfluidics^33,34^. Based on a wettability study, we found that PVA with a hydrolysis degree of 87–90% was more efficient in both the short and long term compared with PVA with a 99+% hydrolysis degree. An explanation for this is that PVA 99+% cannot form a dense and consistent coating on the PDMS surface, which is supported by other studies^27^.

We also investigated the effect of PVA in combination with different oxygen plasma process parameters on modifying the surface properties of the PDMS surfaces. Our results showed that plasma oxidation at high power (100 W) resulted in a more profound and long-lasting surface hydrophilicity. Increasing the power intensity of the oxygen plasma treatment increases the ion bombardment of the PDMS surface, which results in the formation of more γ–OH hydroxyl bonds for surface bonding with the PVA solution^47^. Nevertheless, long and high-power plasma processes (e.g., at 100 W for longer than 5 min) cause excessive heating inside the plasma chamber, which may compromise the thermal integrity of PDMS and lead to cracking of the surface and weak or incomplete bonding^47^. We found that a good balance between the level of the induced surface hydrophilicity and the thermal stability of the PDMS was plasma processing at 100 W for 1 min.

The surface roughness of the PVA-treated PDMS surfaces significantly increased compared with the untreated PDMS. According to Wenzel’s equation^48^, increasing the roughness of a surface enhances the wettability due to the chemistry of the surface. In our case, the increase in the roughness due to the plasma treatment^49^ was further enhanced by the random deposition of PVA^50^. These two combined effects account for the long-lasting hydrophilic effect on the PDMS surfaces (>30 days).

The surface modification method allowed for the high-throughput generation of stable and monodisperse o/w droplets (both with lipids and surfactant). The modification process is not labor intensive; it simply relies on passing a PVA solution through the channels and increasing the throughput production rate of chips. In addition, the treatment remains stable for at least one month after the hydrophilic coating, which highlights the ability of the proposed method to deliver off-the-shelf products that can be maintained without compromising the performance of the microfluidic device.

Moreover, we modified the surface chemistry of specific microchannels on a chip to create partially hydrophilic, partially hydrophobic, channel networks. Treatments based on chemical vapor deposition^19^ and incorporation of an amphiphilic surfactant in the PDMS bulk^20^ are unsuitable in this regard. In a series of experiments, we used our modification method for the selective modification of defined areas, and we constructed devices capable of generating monodispersed w/o/w and o/w/o double emulsions simply by changing which areas were surface modified, which highlights the versatility of our approach.

A major advantage of the proposed approach is that it allows microfluidic chips to be reused by simply flowing nitrogen through the channels to clear them of the aqueous phases. For the removal of more difficult residues in the channels, such as organic dyes, ethanol can be flushed through the channels and followed by a nitrogen flow. This cleaning procedure could be repeated several times without affecting the surface hydrophilicity of the modified PDMS. This approach has considerable advantages in terms of time, effort, and cost effectiveness compared with other approaches. This is particularly relevant for devices used to generate lipid-stabilized droplets based on LbL deposition^22^, which were only used one-time before fouling of the device occurred.

The ability to generate lipid-stabilized o/w droplets in PDMS devices has added significance given the current emergence of droplet microfluidics for the construction of model membranes, including droplet interface bilayers^14,15^, multisomes^51,52^, vesicles^13,53,54^, and artificial cells in general^55^. With respect to droplet generation, lipids are more problematic than surfactants, and many surface modification techniques that are adequate for surfactant-stabilized droplets are inadequate for lipid-stabilized droplets. This work demonstrates that the PVA deposition approach is compatible with lipids.

In conclusion, compared with alternative approaches, the benefits of this method are: (i) the robustness/reusability of the devices, which reduces the cost and effort to make new devices; (ii) the treatment is long lasting (at least 30 days) as opposed to simple UV irradiation and plasma exposure techniques; (iii) the simplicity, convenience, and versatility of our approach, only one type of polymer (PVA) has to be adequately infused and removed; and (iv) the ability to create a spatially patterned microchannel network. The versatility, robustness, and simplicity of this approach has the potential to further accelerate the development of double emulsion technologies and applications. This is particularly important given the increasing potential of double emulsions in academia and industry in uses ranging from the gradual release of cargoes in cosmetics^56^, food science^57^, and pharmaceuticals^56^, to templating vesicles^58^ and polymersomes^59^ with defined architectures for drug delivery purposes and as microreactors for the production of small molecules^52^ and micro- and nanoparticles^60^.
