# Supplementary material for: Hydrophilic surface modification of PDMS for droplet microfluidics using a simple, quick, and robust method via PVA deposition
Source: Microsyst Nanoeng. 2017 Apr 24;3:16091. doi: 10.1038/micronano.2016.91 (PMC6444978; doi:10.1038/micronano.2016.91)
Supplement: Supplementary Information [file micronano201691-s1.pdf]

Microsystems & Nanoengineering (2017) **3**, 16091; doi:10.1038/micronano.2016.91
www.nature.com/micronano

Supplementary file

Hydrophilic surface modification of PDMS for droplet microfluidics using a simple, quick, and robust method via PVA deposition

Tatiana Trantidou[1], Yuval Elani[1,2], Edward Parsons[3] and Oscar Ces[1,2]

Microsystems & Nanoengineering (2017) **3,** 16091; doi:10.1038/micronano.2016.91; Published online: 24 April 2017

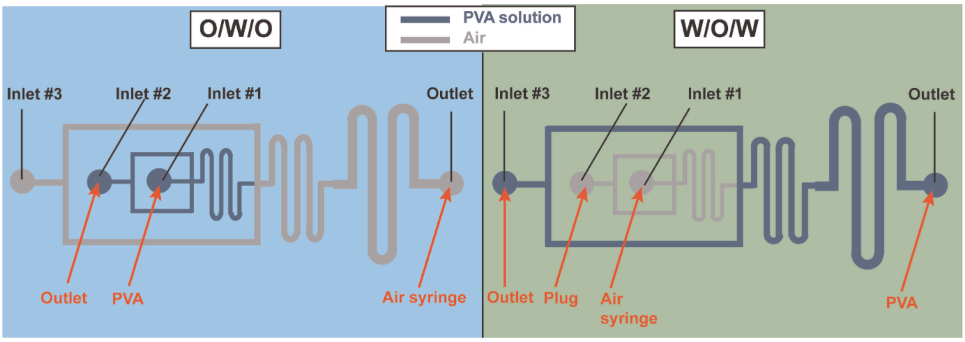

Figure S1 Schematic of the selective PVA treatment of a PDMS microfluidic chip. PVA solution was only passed through the blue channels, leading to selective surface modification. A syringe was applying air at 200 μL min^{-1} through a syringe pump. Then 1 wt% PVA solution is inserted manually in the device for 10 min at room temperature. The solution is removed by blowing nitrogen through the device from the air syringe inlet. Then the device is baked on a hotplate at 110 °C for 15 min. This process is repeated three times for lipid multisome generation.

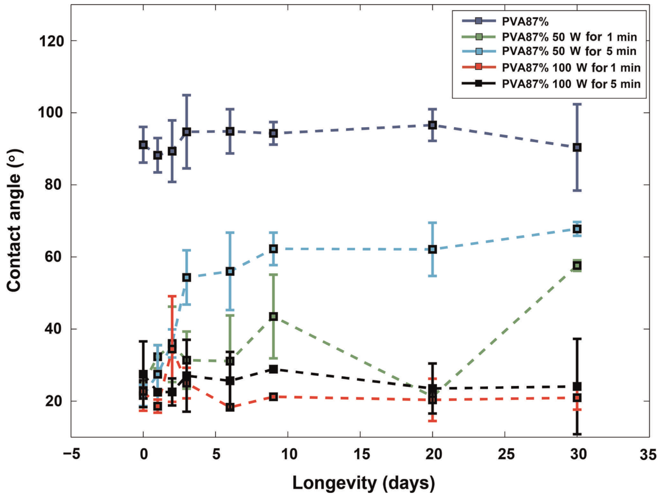

Figure S2 Long-term wettability study of 10 μL water drops on PDMS surfaces treated with PVA (87–90% hydrolysis) under various oxygen plasma conditions.

Supplementary video 1 Lipid stabilized o/w droplet generation on a hydrophilic microfluidic device treated with oxygen plasma (100 W for 1 min) and PVA. The depth of the channels was 80 μm. O/w droplets were produced at flow rates of 1 μL min^{-1} and 15 μL min^{-1} respectively.

Supplementary video 2 Oil/surfactant droplet generation at a microfluidic chip treated with oxygen plasma (100 W for 1 min) and PVA. The depth of the channels was 80 μm. O/w droplets were produced at flow rates of 1 μL min^{-1} and 5 μL min^{-1} respectively.

Supplementary video 3 Lipid stabilized o/w droplet generation at a microfluidic chip that was treated with oxygen plasma (100 W for 1 min) and PVA 30 days previously. The device was stored in standard room conditions. The depth of the channels was 80 μm. O/w droplets were produced at flow rates of 1 and 15 μL min^{-1} respectively.

Supplementary video 4 O/w/o double emulsion generation at a microfluidic chip that was selectively treated with oxygen plasma (100 W for /1 min) and PVA. The depth of the channels was 100 and 200 μm at the first and second flow-focussing junctions respectively. O/w/o double emulsions were produced at flow rates of 1, 8, and 20 μL min^{-1} respectively.

Supplementary video 5 W/o/w double emulsion generation at a microfluidic chip that was selectively treated with oxygen plasma (100 W for 1 min) and PVA. The depth of the channels was 100 and 200 μm at the first and second flow-focussing junctions respectively. W/o/w double emulsions were produced at flow rates of 1, 5, and 12.5 μL min^{-1} respectively.

[1]Department of Chemistry, Imperial College London, London SW7 2AZ, UK; [2]Institute of Chemical Biology, Imperial College London, London SW7 2AZ, UK and [3]London Centre for Nanotechnology, University College London, London WC1E 6BT, UK.
Correspondence: Tatiana Trantidou (t.trantidou@imperial.ac.uk) and Oscar Ces (o.ces@imperial.ac.uk)

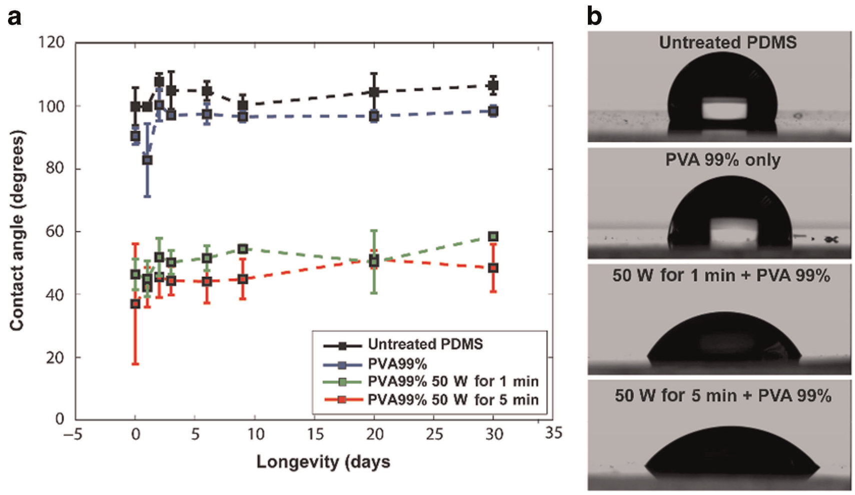

Figure S3 Contact angle measurements of PDMS surfaces treated with PVA (99+% hydrolysis) as a function of time. **(a)** Untreated and PVA (99+% hydrolysis) treated PDMS under various oxygen plasma conditions. **(b)** 10 μL water affinity drops on PDMS surface. As expected, the PVA treatment had practically no effect on native hydrophobic PDMS without previous oxygen plasma treatment (99.87 ± 6.02° for untreated PDMS vs. 91.10 ± 4.96° for PVA treated PDMS).

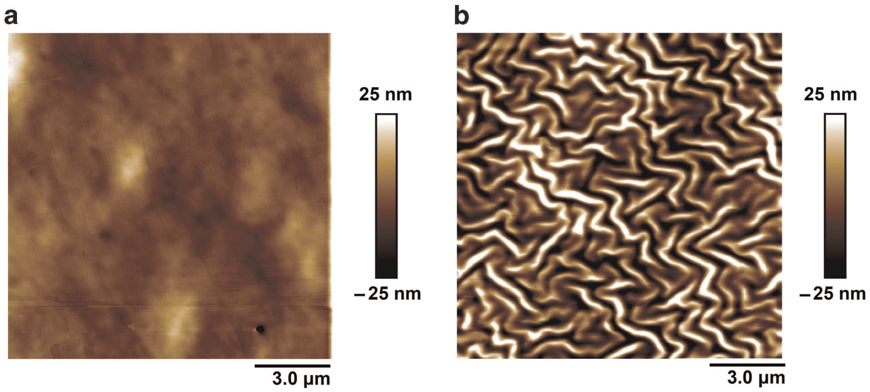

Figure S4 AFM images of (**a**) an untreated PDMS surface and (**b**) a plasma oxidised (100 W for 1 min) PDMS surface followed by treatment with PVA (87–90% hydrolysis). Measurements were taken in 50 μL of aqueous solution (72% v/v milli-Q water, 14% v/v ethanol and 14% v/v glycerol). Both images are 12 × 12 μm.

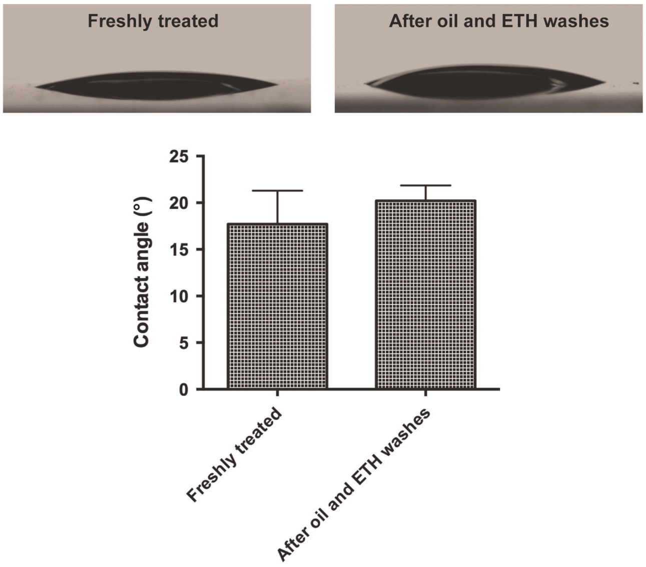

Figure S5 Top: 10 μL water affinity drops on PVA-treated PDMS surfaces freshly after treatment (left) and after covering the surface with oleic acid for 5 min and washing it with ethanol (right). Bottom: Corresponding statistical study (paired *t*-test, $N = 4$ samples) of contact angle measurements indicating no significant ($P < 0.05$) difference between the two groups. Error bars indicate the standard deviation of the means.
